# Advanced 3D-DXA insights into bone density changes in hyperparathyroidism

**DOI:** 10.1007/s40200-024-01487-3

**Published:** 2024-08-29

**Authors:** Francesco Saverio Guerra, Nicola Palladino, Renaud Winzenrieth, Giuseppe Guglielmi

**Affiliations:** 1 Department of Clinical and Experimental Medicine, Foggia University School of Medicine, Foggia, Italy; 2 3D-Sharper Medical, Barcelona, Spain; 3 Department of Radiology, Hospital IRCCS Casa Sollievo Della Sofferenza, San Giovanni Rotondo, Viale L. Pinto 1, 71121 Foggia, Italy; 4 Radiology Unit, ‘‘Dimiccoli’’ Hospital, Viale Ippocrate, 15, 70051 Barletta, Italy

**Keywords:** Hyperparathyroidism, Mineral density, Trabecular bone, Cortical bone, 3D-DXA

## Abstract

**Objectives:**

Primary hyperparathyroidism (PHPT) is a disorder marked by chronic parathyroid hormone hypersecretion, which affects bone turnover and remodelling processes. With a loss of bone density and an increase in bone porosity, the cortical compartment is most severely impacted. The study's goal is to assess PHPT's effects on the volumetric bone mineral density (vBMD) of the femur's trabecular compartment as well as the vBMD and thickness of the cortical bone.

**Methods:**

This is a retrospective case–control study, valuating age, biochemical doses, anthropometric measurements, and bone measurements. Between 2011 and 2016, 74 Caucasian Italian women and men with PHPT were sought out. Biochemical analyses were added to bone mineral density (BMD) values found in the lumbar spine and femoral neck. Proximal femur parameters such as cortical and trabecular volumetric (v) BMD, cortical thickness (CTh) and surface (s) BMD were analyzed by 3D-DXA software (3D-SHAPER Medical, Spain).

**Results:**

The findings showed a negative correlation between PHPT patients and controls, which was equally affecting the cortical and trabecular compartments. This correlation was especially evident in the areal BMD (aBMD) and vBMD measurements. Nonetheless, no appreciable correlation was found between the cortical level and the thickness of the cortical bone.

**Conclusions:**

Parathormone (PHT) levels had an adverse effect on the cortical, trabecular volumetric density in this investigation, as was expected. Cortical thickness, however, is unaffected significantly. The literature and these findings are consistent.

## Introduction

A common endocrine condition called primary hyperparathyroidism (PHPT), which affects calcium metabolism, causes mild or asymptomatic hypercalcemia and high or inappropriately normal parathormone (PTH) levels [1, 2]. However, normocalcemic individuals also exhibit PHPT. Normocalcemic hyperparathyroidism (nPHPT) is a form of primary hyperparathyroidism (PHPT) where the patient has normal levels of calcium in the blood but elevated levels of parathyroid hormone (PTH). This condition can be challenging to diagnose because it lacks the classic symptom of hypercalcemia (high blood calcium levels) typically associated with primary hyperparathyroidism. Normal serum calcium levels and ionised calcium levels, as well as the absence of secondary hyperparathyroidism, are characteristics of normocalcemic nPHPT [3].

Secondary hyperparathyroidism is a condition where increased PTH secretion is a compensatory response to other physiological conditions. To accurately diagnose nPHPT, secondary causes must be ruled out. The most common causes include vitamin D deficiency, renal insufficiency, malabsorption syndromes, and other metabolic disorders. Diagnosing normocalcemic hyperparathyroidism requires careful exclusion of all potential secondary causes of elevated PTH. A comprehensive laboratory evaluation that includes tests for vitamin D levels, renal function, phosphate levels, and other relevant metabolic parameters is essential [3].

PHPT results from inappropriate PTH secretion from one or more of the four parathyroid glands. The majority of PHPT patients (80%) have a single adenoma, although in 20% of cases, PHPT patients also have multiple adenomas (10%), four gland hyperplasia (10%), or parathyroid cancer (1%) [3].

One of the key issues for PHPT patients is their skeletal health [2]. One characteristic of PHPT is high bone turnover, which favors bone resorption over bone formation [2, 4]. A high or excessive excretion of PTH is associated with this fast bone turnover. Despite the substantial bone turnover anticipated as a result of this, just a slight decline in bone mineral density (BMD) is often seen. Contradictions do, however, arise amongst the various skeleton locations [4].

While a relative bone preservation is seen at the spine, a more pronounced impairment is typically seen in cortical sites like the radius, and to a lesser extent, at the femur [3]. Additionally, it has been demonstrated that trabecular bone impairment can also happen in non-weight-bearing sites, emphasising the protective nature of mechanical loading on trabecular bone. Finally, the duration and severity of the PTH excess determine how severe this bone impairment is [5].

Dual Energy X- Ray Absorptiometry (DXA), which measures areal BMD (aBMD) expressed in g/cm2, is the gold standard imaging tool for evaluating bone health in patients with PHPT. This diagnostic procedure is particularly efficient, especially at the level of the lumbar spine and the femur, thanks to its advantages of being non-invasive, quick, and adaptable to different populations [6, 7]. The 2D examination, however, only gives an average value for the area of interest under consideration and does not distinguish between the cortical and trabecular bones.

In PHPT patients, it is crucial to be able to evaluate cortical and trabecular bones separately. Quantitative computed tomography (QCT) with a small voxel size (≤ 1 mm per side) is the current “gold standard” to examine the macrostructure of the femur, but it has a high effective radiation dose (approximately 2 to 5 mSv) and cost [8]. Volumetric dual-energy X-ray absorptiometry (VXA) uses a commercially available DXA system to reconstruct the proximal femur from four DXA scans delivering an effective radiation dose of 0.04 mSv. VXA is capable of generating a variety of 3D geometric and structural measurements that are highly correlated with QCT in elderly subjects in vivo [8]. Moreover, the VXA measurements can be made with a commercially available DXA device at a very low radiation dose. DXA is inexpensive, has a very low radiation dose (effective dose of less than 0.01 mSv), and has been shown to be cost-effective for the management of bone disease [8]. Nevertheless, as a measure of the structural integrity of bone, DXA has limitations. DXA is a 2D projection measurement of a 3D object, which limits the geometric and structural information that can be derived from a DXA exam and may, in turn, limit its ability to predict fracture risk. This may account, in addition to the propensity to fall, for the fact that that more than half of women who suffer a hip fracture do not have low aBMD as measured by DXA [8].

To overcome DXA and QCT limitations, DXA-based 3D modelling methods have recently been introduced [9]. Briefly, 3D-DXA uses a statistical 3D model of the proximal femur's shape and density created using a database of QCT scans. I In order to create a 3D patient-specific QCT-like model of the proximal femur, this model is registered onto the patient's hip DXA scan. Following segmentation, the cortical compartment is described by its thickness (CtTh in mm), volumetric bone mineral density (vBMD in mg/cm3), and surface bone mineral density (sBMD in mg/cm2), calculated by multiplying the vBMD and the CtTh at each vertex of the femoral surface of the 3D model), while the trabecular compartment is described by its vBMD (in mg/cm3). The accuracy and precision of 3D-DXA have been assessed in previous work [9].

3D-DXA has been used to assess how osteoporotic treatments affect bone. Teriparatide, a PTH analogue, has been demonstrated to have hip-related effects in osteoporotic women by a number of authors, underlining PTH's catabolic effects on cortical bone and its anabolic effects on trabecular bone [10].

3D-DXA has also been recently used to evaluate the bone health of PHPT patients [11]. In this study, the investigators compared 40 hypercalcemic PHPT patients to 40 healthy controls who were age and gender matched. Authors found no significant differences in trabecular vBMD, but they did find significant impairments in all cortical parameters at the total femur, ranging from 3.7% to 8.9% when compared to controls [12]. They also suggested that PHPT may have a regional effect because the trochanteric area showed more severe impairments than the femoral neck, although they did not quantify this effect.

The aim of the present study is to investigate regional effect of PTH excess on cortical and trabecular bones in nPHPT patient using a DXA-based 3D modelling approach.

## Material and methods

### Study design and participants

This case–control study uses retrospective data (2011–2016) from Caucasian men and women from the Radiology Unit and interdisciplinary team of the IRCCS Casa Sollievo della Sofferenza in San Giovanni Rotondo, Italy. Suitable candidates for inclusion were determined to be subjects with at least one hip DXA scan and a concomitant biological dosimetry (one year after the first DXA exam).

All patients who displayed a largely uniform clinical profile were included in the nPHPT group. Particularly, elevated PTH levels, elevated serum and urine calcium levels were seen in all PHPT patients. Renal function was not compromised in the patients, nevertheless. On the other hand, the majority of patients' VitD25(OH) readings fell below the threshold.

Phosphatase and the other values that were checked were within the normal range. Subjects qualified for participation in the control group provided they met two criteria: 1) no prior low energy fracture; and 2) normal levels of blood ionised calcium, serum total calcium, phosphate, creatinine, and PTH according with the normal range of our laboratory of clinical biochemistry.

Patients were excluded if they had a body mass index (BMI) of 40 or higher, early menopause for women, bilateral hip replacements, anti-resorptive medications taken within the last five years, or co-morbid conditions that affect bone metabolism.

## Outcomes measures

### Anthropometric measures

Medical records and the hospital's DXA database were used to acquire demographic data. The operator manually measured the subjects' weight (kg) and height (m) using the same scale and stadiometer, respectively. Weight divided by height squared was used to determine the body mass index (BMI, kg/m^2^).

### Biological dosimetry

After fasting for the previous night, venipuncture was used to collect venous blood samples. The measurement of a few significant values for the remodelling and metabolism of bones served as the basis for the biochemical analysis. Specifically, calcium (mg/dL, corrected for serum albumin) and PTH level (between 12–88 pg/mL) were measured using conventional automated laboratory techniques. For calcium, normal values fall between 8.8 and 10.2 mg/dL, for vitamin D25(OH), between 20 and 40 ng/mL, and for creatinine clearance dosage, between 30 and 120 mL/min.

### Areal bone mineral density

Using an iDXA densitometer (GE Healthcare, USA), evaluations of areal BMD (aBMD, in g/cm^2^) at the spine (L1-L4), total hip, and femoral neck were made. In accordance with the guidelines provided by the International Society of Clinical Densitometry [13], one trained operator analysed all DXA scans using Encore software (GE Healthcare, USA). To evaluate performance, daily quality assurance data from the DXA scanner were collected. Following the criteria for osteoporosis established by the World Health Organisation, densitometric osteoporosis was defined as a T-Score lower than or equal to -2.5 at the spine (L1-L4), total hip, or femoral neck [9].

### 3D-DXA modelling

Trabecular and cortical bones were evaluated in three dimensions using the 3D-Shaper software (version 2.10.4, 3D-SHAPER Medical, Barcelona, Spain). In essence, the software builds a subject-specific QCT-like model of the proximal femur from a standard hip DXA scan. Details of 3D-DXA can be found elsewhere [9]. The current study included the following variables: cortical volumetric BMD (cortical vBMD in mg/cm^3^), trabecular volumetric BMD (trabecular vBMD in mg/cm^3^), integral (cortical + trabecular) volumetric BMD (integral vBMD in mg/cm^3^), and cortical surface BMD (cortical sBMD in g/cm^2^) calculated as the multiplication of the Cortical Thickness (CtTh expressed in cm) and the cortical vBMD (expressed in g/cm^3^). All measurements were calculated at the neck, trochanter, and shaft as well as at the total femur. These regions are identical to those identified by DXA scans.

### Statistical analysis

Results for all parameters were expressed as mean and standard deviation (SD) or as a median and 95% confidence intervals (CI) after being verified to have a normal distribution.

Depending on the data's normality, Wilcoxon signed ranks tests or Student's t-tests were used to compare the two groups (PHPT vs. control group). The occurrence of PHPT was further investigated in relation to clinical parameters, aBMD, and 3D parameters using univariate analyses and multivariate regression models (using stepwise variable selection). Areas under the receiver operating characteristic curves (AUC) were used to evaluate the discriminating capacity of the measured parameters, and odds ratios (OR) were computed to assess the degree of association between independent variables and the existence of PHPT. The threshold for statistical significance was set at *P* = 0.05 for all two-tailed inferential tests. R software (v3.6.0) was used to do the statistical analysis. Integral vBMD, trabecular vBMD, cortical sBMD, cortical vBMD, and CtTh differences between PHPT and control groups were estimated on a three-dimensional (3D) spaces.

To further analyse the anatomical distribution of the differences between the PHPT and control group in the cortical and trabecular compartments, an average 3D model of the proximal femur was computed for each group using registration techniques. These average models were used to create 3D maps, mid-coronal slices, and slices at the neck, the trochanteric, and lower shaft regions showing the differences between the two groups.

## Results

### Subject characteristics

After application of inclusion and exclusion criteria, 74 subjects (88% of women) were included in the study. Among them, 40 were included in the nPHPT group with a mean age and BMI of 59.1 ± 13.0 years and 29.2 ± 6.5 kg/m^2^ respectively. 34 subjects were included in the control group (mean age and BMI of 60.4 ± 11.9 years and 27.9 ± 8.5 kg/m^2^ respectively). Subject characteristics included in each group are included in Table 1. No differences in age (*P* = 0.65), height (*P* = 0.78), weight (*P* = 0.59), BMI (*P* = 0.47), or gender (*P* = 0.18) were found between the nPHPT and control groups. PHPT subjects had significant higher PTH level when compared to control subjects (42.7 vs 111.5 pg/ml, *P* < 0.01), lower VitD level (22.6 vs 29.6 ng/*ml*, *P* < 0.01), higher urinary calcium in 100ml (10.6 vs 16.0, *P* < 0.05) and higher blood calcium (9.3 vs 10, *P* < 0.01) while no differences were observed for phosphate and creatinine clearance (all *P* > 0.05).

**Table 1:** Descriptive statistics for the group under study

	**Control** n = 34	**PHPT** n = 40	**p**
Gender (M/W)	(6/28)	(3/37)	ns
Age (years)	60.4 ± 11.9	59.1 ± 13.0	ns
Weight (kg)	72.3 ± 23.9	74.9 ± 18.4	ns
Height (cm)	160.7 ± 8.6	160.1 ± 8.5	ns
BMI (kg/m2)	27.9 ± 8.5	29.2 ± 6.5	ns
Prevalent fracture (n)	2/34	6/40	ns
**Biochemical Dosimetry**			
PTH pg/ml	42.7 [37.5—48.7]	111.5 [81.1—151.2]	**
VitD25(OH) ng/ml	29.6 [23.7—37.0]	22.6 [21.0—27.6]	*
Urinary calcium in 100 ml mg/dl	10.6 [7.6—14.4]	16.0 [10.8—19.9]	*
Blood Calcium mg/dl	9.3 [8.9—9.5]	10.0 [9.7—10.4]	**
Creatinine Clearance ml/mn	85.4 [79.8—101.6]	103.5 [82.9—119.7]	ns
Phosphatase Ul/l	70.0 [58.2—87.3]	72.2 [65.5—91.0]	ns
**2D DXA**			
aBMD Total (g/cm2)	0.938 ± 0.167	0.859 ± 0.153	*
aBMD Neck (g/cm2)	0.842 ± 0.151	0.794 ± 0.145	ns
aBMD Trochanteric (g/cm2)	0.759 ± 0.147	0.696 ± 0.144	ns
aBMD Shaft (g/cm2)	1.129 ± 0.212	1.025 ± 0.183	*
**3D parameters**			
**Total Femur**			
Integral vBMD (mg/cm3)	307.1 ± 69.2	272.8 ± 57.2	*
Cortical sBMD (mg/cm2)	157.7 ± 32.5	143.3 ± 28.0	*
Trabecular vBMD (mg/cm3)	151.9 ± 47.2	131.8 ± 43.6	ns
Cortical vBMD (mg/cm3)	829.3 ± 116.8	766.1 ± 92.3	*
Cortical Thickness (mm)	1.891 ± 0.180	1.859 ± 0.199	ns
**Neck**			
Integral vBMD (mg/cm3)	319.2 ± 62.0	290.0 ± 57.2	*
Cortical sBMD (mg/cm2)	123.4 ± 22.8	113.4 ± 21.1	ns
Trabecular vBMD (mg/cm3)	189.1 ± 48.9	170.1 ± 49.1	ns
Cortical vBMD (mg/cm3)	807.2 ± 93.9	749.9 ± 77.0	**
Cortical Thickness (mm)	1.555 ± 0.16	1.543 ± 0.194	ns
**Trochanteric**			
Integral vBMD (mg/cm3)	224.4 ± 51.5	198.0 ± 45.3	*
Cortical sBMD (mg/cm2)	148.5 ± 29.6	134.8 ± 24.5	*
Trabecular vBMD (mg/cm3)	130.5 ± 38.8	112.5 ± 36.3	*
Cortical vBMD (mg/cm3)	687.6 ± 89.0	634.0 ± 76.2	**
Cortical Thickness (mm)	1.815 ± 0.194	1.784 ± 0.196	ns
**Shaft**			
Integral vBMD (mg/cm3)	365.7 ± 80.4	321.4 ± 65.1	*
Cortical sBMD (mg/cm2)	246.5 ± 48.2	222.2 ± 42.0	*
Trabecular vBMD (mg/cm3)	152.9 ± 53.6	130.4 ± 48.9	ns
Cortical vBMD (mg/cm3)	891.4 ± 110.7	821.6 ± 91.8	**
Cortical Thickness (mm)	2.818 ± 0.301	2.751 ± 0.321	ns

### DXA measurements

**Fig. 1:**
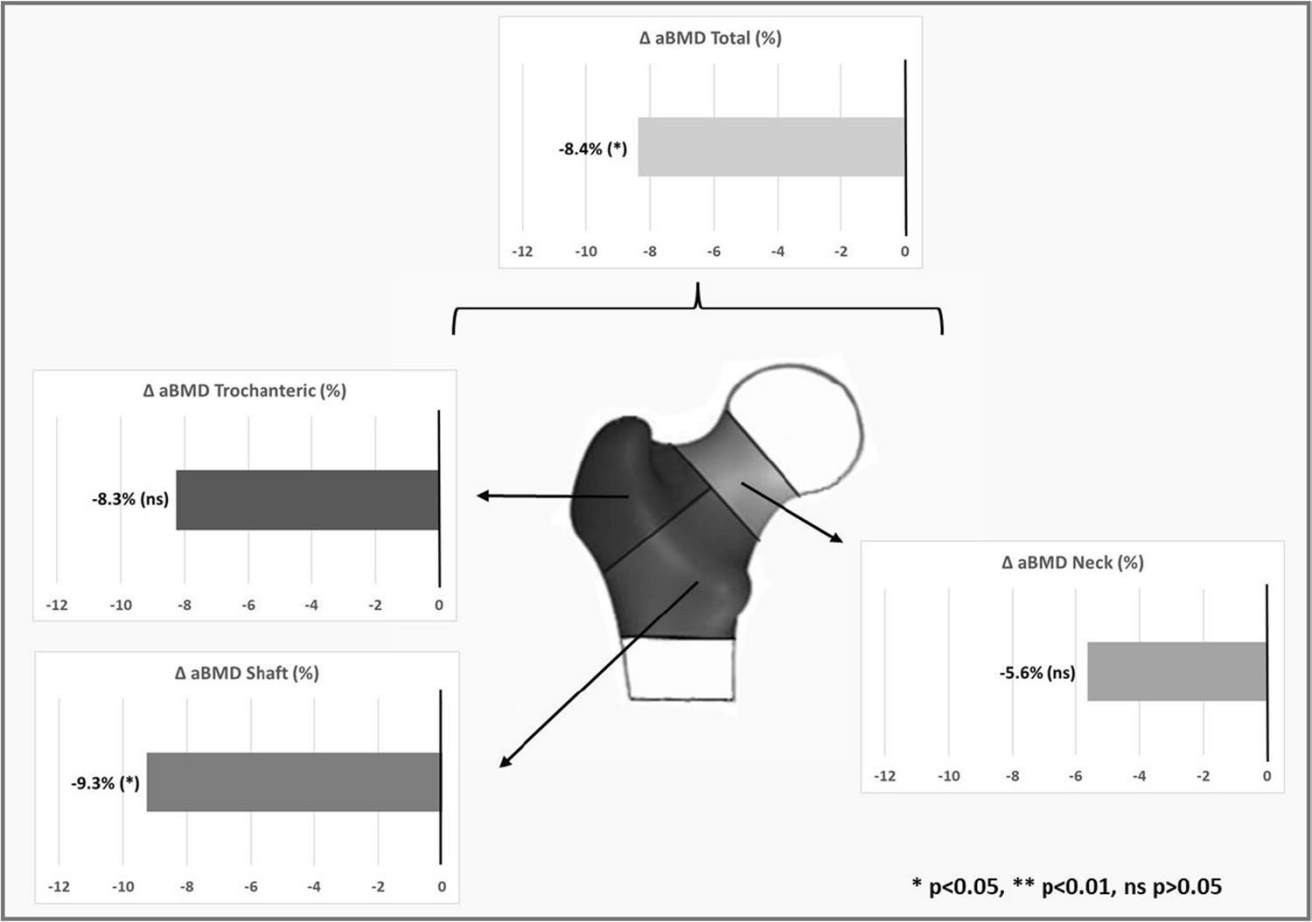
3BMD^*^ differences (∆) in % (nPHPT^**^ subjects against controls) at the total femur, neck, trochanteric and shaft regions. ^*^aBMD (areal bone mineral density); ^**^PHPT (Primary hyperparathyroidism)

Subjects in the nPHPT group had lower aBMD at total femur (0.859 vs 0.938, *P* = 0.04) and at the shaft (1.025 vs 1.129, *P* = 0.026) while no significant differences were observed at the neck (*P* = 0.17) or at the trochanteric region (*P* = 0.07) (Fig. 1).

### 3D measurements

**Fig. 2:**
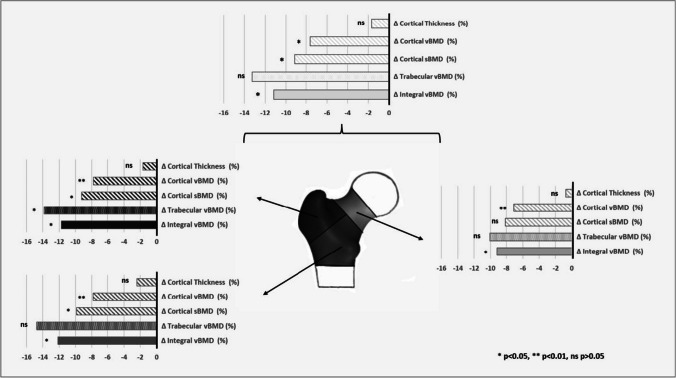
3D parameter differences (∆) in % (nPHPT^*^ subjects against controls) at the total femur, neck, trochanteric and shaft regions. ^*^PHPT (Primary hyperparathyroidism)

Integral vBMD in the nPHPT group was 11.2% lower at the total hip (Fig. 2) as compared to the control group (*P* = 0.05). Cortical sBMD in the nPHPT group was considerably lower than in the control group (-9.1%, *P* = 0.04). While no difference was seen for CtTh (*P* = 0.5), this difference is associated with a lower cortical vBMD (-7.6%, *P* = 0.01). Trabecular vBMD did not significantly differ between the nPHPT and control groups, despite being lower in the PHPT group (*P* = 0.06).

No differences between the nPHPT and control groups were seen for CtTh (all *P* > 0.35, Fig. 1) regardless of the region of interest (neck, trochanter, or shaft), while the nPHPT group showed significantly lower values for cortical vBMD (all *P* = 0.01). As shown by declines of -7.1%, -7.8%, and—7.8% at the neck, trochanteric, and shaft areas, respectively, cortical vBMD impairment is homogenous in amplitudes in the various regions (Fig. 1). When compared to the control group, the nPHPT group had significantly lower cortical sBMD at the trochanteric (-9.2%, *P* = 0.03) and shaft (-9.9%, *P* = 0.02) regions (Fig. 1).

**Fig. 3:**
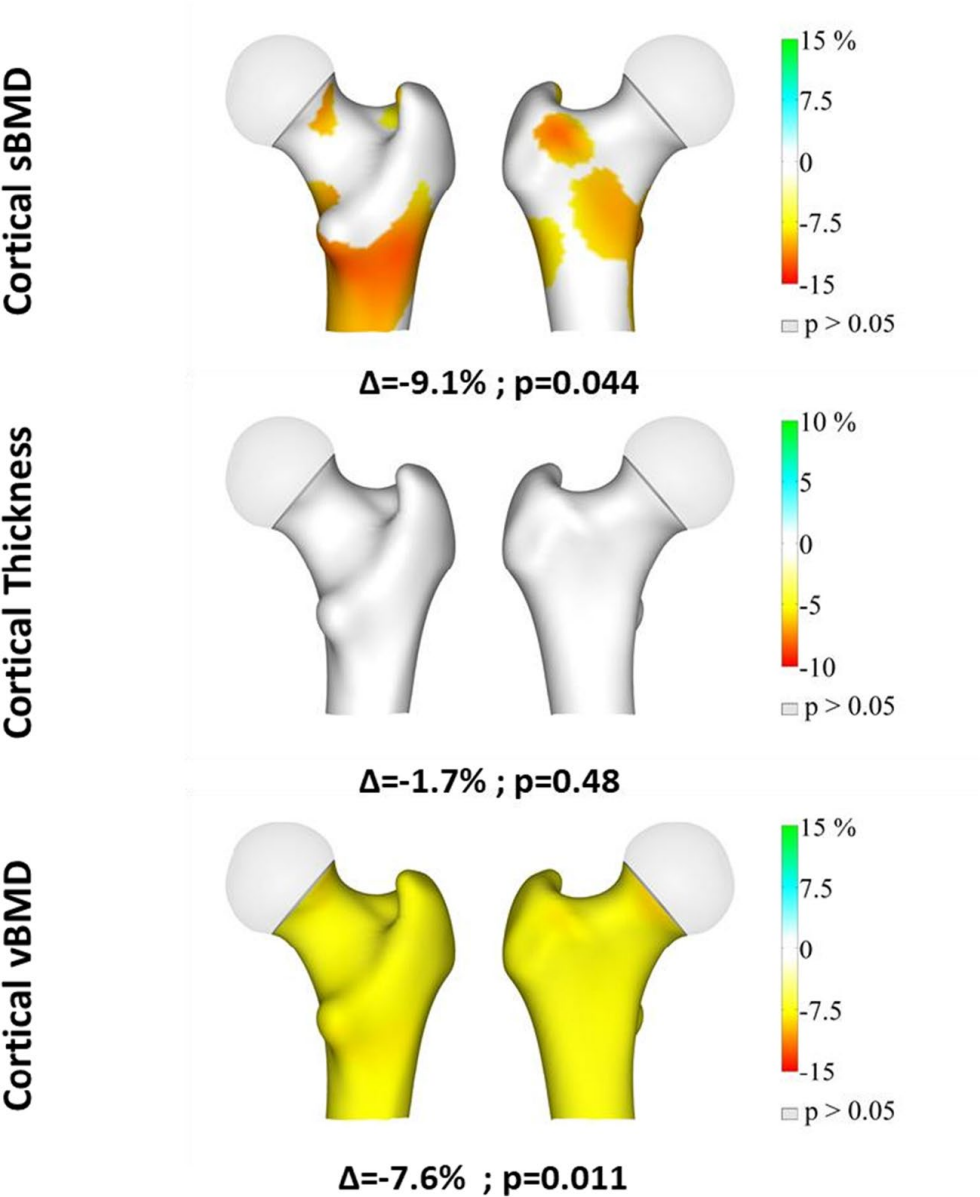
Anatomical distribution of percentage differences (nPHPT^*^ subjects against controls) for the cortical parameters. Positive differences are in blue-green color while negative differences are in yellow–red color. Only significant differences are displayed. Grey color represents regions with no significant differences. ^*^PHPT (Primary hyperparathyroidism)

**Fig. 4:**
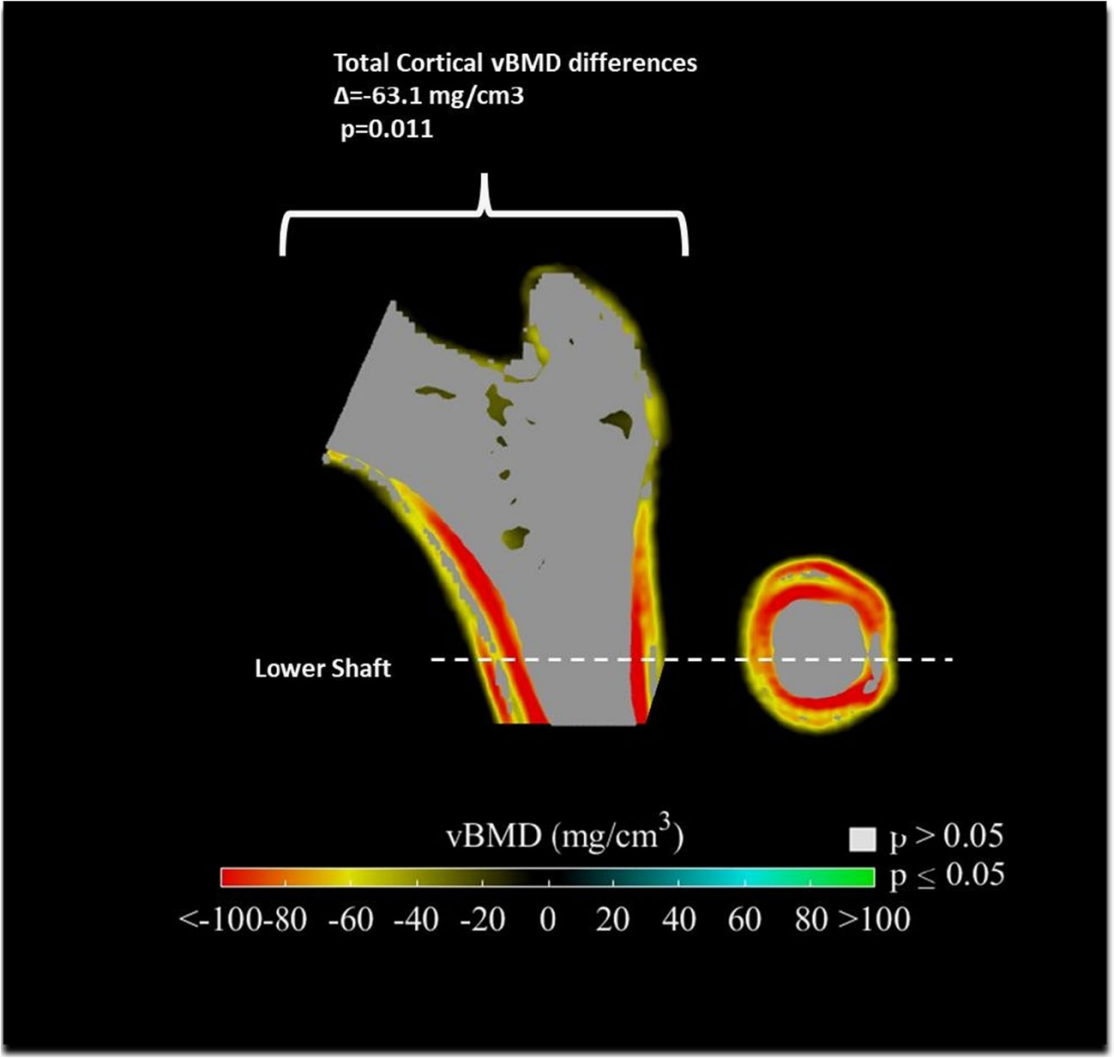
Anatomical distribution of vBMD^*^ differences (nPHPT^**^ subjects against controls) in the mid-coronal and lower shaft cross sections. Positive differences are in blue-green color while negative differences are in yellow–red color. Only significant differences are displayed. Grey color represents regions with no significant differences. ^*^vBMD (volumetric bone mineral density); ^**^PHPT (Primary hyperparathyroidism)

Figure 3 shows the anatomical distributions of the differences between the groups at the cortex. Cortical vBMD was found to be uniformly impaired in the nPHPT group (Fig. 3, bottom). Cortical sBMD was found to be reduced at the shaft (Fig. 3, top), while no significant differences were found in cortical thickness (Fig. 3, middle). The marked differences in cortical vBMD (especially at the shaft) can also be seen in Fig. 4 (mid-coronal and lower shaft cross sections), while no difference in vBMD was found in the trabecular compartment.

## Discussion

In the present study, we have evaluated bone pattern differences existing between nPHPT subjects and healthy control subjects using DXA-based 3D modelling technique.

The main outcome of our study is that:

1. The aBMD at the femoral neck and total femur appeared markedly reduced in the patients with nPHPT compared to the control group.
2. 3D-DXA analysis showed a significant impairment of the cortical compartment in the nPHPH group, while no significant difference was found in the trabecular compartment.
3. The cortical impairment in nPHPT subjects is mainly linked to an impairment of the cortical vBMD and to a lesser extent to the cortical thickness.
4. Although not significant, a lower trabecular vBMD was observed in the nPHPT when compared to the control group. In addition to these findings, DXA-based 3D modelling approach provides new insight of PHPT induced bone pattern disorders at a major site of the central skeleton.

Significant cortical vBMD deficits were seen in all sub-regions as well as the total femur in nPHPT individuals. The catabolic impact of high PTH on the cortex has been seen at the radius and hip using DXA [14–16] and HrpQCT [13, 17], which is consistent with this study. The pattern of cortical damage seen in this investigation was slightly different from what reported by Gracia et colleagues [11]. While the opposite was seen in the study by Bandeira et al. [18], we saw a significant impairment of the cortical vBMD without a significant impairment of the cortical thickness. This may be related to the patient profiles, which were normocalcemic in the current study and hypercalcemic in the previous studies [9, 19, 20].

The number of osteoclasts and osteoblasts is increased in PHPT patients, but their activity is inhibited. The activation frequency of individual bone remodelling units is increased while the ultimate resorption depth is decreased and the wall thickness resulting in a shallower remodelling process. However, the formative surface is grown while the rate of calcification and bone formation has dropped. In spite of trabecular bone having a normal bone mass density, this can increase the risk of microstructural fractures and lower bone quality. The same process at endocortical bone enhanced bone porosity, and heightened reabsorption at endocortical bone surface led to permanent cortical thinning [21, 22]. In the past, PHPT was thought to primarily cause cortical bone loss [23, 24], but more recent research shows that PHPT patients have a considerably increased risk of fracture in sites with trabecular bone predominance, such as the vertebrae, distal forearm, rib, and pelvis [5, 24].

When compared to the control group, the 3D mappings revealed a uniformly lower cortical vBMD over the proximal femur. A measure of cortical bone strength, cortical sBMD mapping showed no abnormalities in the femoral neck but more localized deficiencies, mainly in the inter-trochanteric area, which could potentially result in an increased risk of fracture in this area. To verify this premise, more research is required.

PTH excess also has a deleterious effect on the trabecular compartment, albeit to a smaller degree than on the cortical bone and to no significant degree. This result is consistent with studies from the literature [5, 9, 19, 20, 25] that point to a lessened impact of PTH excess at bone sites that are mostly trabecular and weight-bearing bone sites. However, in hypercalcemic PHPT subjects, some authors have also noticed a trend of trabecular impairment using the same 3D technology [9, 11, 15].

Currently, the only treatment that can be instituted in these patients is the administration of monthly or weekly doses of VitD, in addition to recommending a calcium-rich, low-sodium diet and physical activity. This type of treatment is recommended both in patients who have had parathyroidectomy surgery and those who have not yet had the procedure. The dose is what changes.

### Strengths and limitations

Since there was a control group in this study, we used the raw 2D-DXA data and 3D-DXA software to analyze differences between PHPT patients and controls at the level of the cortical compartment and at the level of the cortical group. The predominance of the female sex is unquestionably higher in both groups, which is further supported by the fact that women are three times more likely than men to have hyperparathyroidism. Patients with PHPT were not as homogeneous as those in the control group, which is undoubtedly one of our study's limitations; in fact 91 patients were recruited in the study group, while 53 in the control group. In addition, with this study we have assessed how PHPT impairs the trabecular and the cortical compartment, but what is actually the catabolic mechanism and how it differs action on the two compartments is still unknown. A further limitation is represented by the quantitative analysis of the reduction on the trabecular compartment, while it would have been useful to perform a second analysis on the quality of trabecular bone, using also the TBS.

Finally, the minimal effect of PHPT on cortical bone thickness represents another limitation that precluded us from evaluating the predictivity of fracture risk in patients with PHPT. Perhaps in the future it could be useful to perform a second analysis, introducing among the parameters to be used for statistical adjustments, the values of VitD25 (OH) and any ongoing therapies.

## Conclusions

The results indicate that patients with PHPT have a significant reduction in aBMD at the hip level, particularly at the localizations highlighted in the 3D map. This is also confirmed by vBMD measurements both in the cortical compartment and at the level of the bony trabeculation. There is no correlation with the thickness of the cortical bone. It would be useful in the future to carry out studies including a qualitative analysis of the trabecular bone and including additional biochemical values (VitD25OH, serum Caclium, etc.) to possibly express a judgment on the potential increased risk of fractures.

## Data Availability

The study data are archived in the “Casa Sollievo della Sofferenza Hospital” database of San Giovanni Rotondo(FG) Italy.
